# Comprehensive investigations revealed consistent pathophysiological alterations after vaccination with COVID-19 vaccines

**DOI:** 10.1038/s41421-021-00329-3

**Published:** 2021-10-26

**Authors:** Jiping Liu, Junbang Wang, Jinfang Xu, Han Xia, Yue Wang, Chunxue Zhang, Wei Chen, Huina Zhang, Qi Liu, Rong Zhu, Yiqi Shi, Zihao Shen, Zhonggang Xing, Wenxia Gao, Liqiang Zhou, Jinliang Shao, Jiayu Shi, Xuejiao Yang, Yaxuan Deng, Li Wu, Quan Lin, Changhong Zheng, Wenmin Zhu, Congrong Wang, Yi E. Sun, Zhongmin Liu

**Affiliations:** 1 Shanghai Institute of Stem Cell Research and Clinical Translation, Shanghai East Hospital, School of Medicine, Tongji University, Shanghai, China; 2 Key Laboratory of Spine and Spinal Cord Injury Repair and Regeneration of Ministry of Education, Orthopedic Department of Tongji Hospital, School of Medicine, Tongji University, Shanghai, China; 3 Department of Health Statistics, Second Military Medical University, Shanghai, China; 4 Key Laboratory of Special Pathogens and Biosafety, Wuhan Institute of Virology, Center for Biosafety Mega-Science, Chinese Academy of Sciences, Wuhan, Hubei China; 5 University of Chinese Academy of Sciences, Beijing, China; 6 Department Endocrinology & Metabolism, Shanghai Fourth People’s Hospital, School of Medicine, Tongji University, Shanghai, China

**Keywords:** Immunology, Transcriptomics

## Abstract

Large-scale COVID-19 vaccinations are currently underway in many countries in response to the COVID-19 pandemic. Here, we report, besides generation of neutralizing antibodies, consistent alterations in hemoglobin A1c, serum sodium and potassium levels, coagulation profiles, and renal functions in healthy volunteers after vaccination with an inactivated SARS-CoV-2 vaccine. Similar changes had also been reported in COVID-19 patients, suggesting that vaccination mimicked an infection. Single-cell mRNA sequencing (scRNA-seq) of peripheral blood mononuclear cells (PBMCs) before and 28 days after the first inoculation also revealed consistent alterations in gene expression of many different immune cell types. Reduction of CD8^+^ T cells and increase in classic monocyte contents were exemplary. Moreover, scRNA-seq revealed increased NF-κB signaling and reduced type I interferon responses, which were confirmed by biological assays and also had been reported to occur after SARS-CoV-2 infection with aggravating symptoms. Altogether, our study recommends additional caution when vaccinating people with pre-existing clinical conditions, including diabetes, electrolyte imbalances, renal dysfunction, and coagulation disorders.

## Introduction

The COVID-19 pandemic has profoundly affected humanity. The development of COVID-19 vaccines in various forms has been underway in an unprecedented and accelerated manner. Despite some uncertainties regarding potential consequences, large-scale vaccinations are taking place in many countries. There have been different COVID-19 vaccines developed, including inactivated viral particles, mRNA vaccines, adenoviral-based vaccines, and etc.^1–5^. Historically, vaccine research has been focused on whether or not vaccination could generate neutralizing antibodies to protect against viral infections, whereas short-term and long-term influences of the various newly developed vaccines to human pathophysiology and other perspectives of the human immune system have not been fully investigated.

With the development of large-scale single-cell mRNA sequencing (scRNA-seq) technology, systematic investigation of people’s immune system function with precision became possible, primarily through scRNA-seq of peripheral blood mononuclear cells (PBMCs). During the COVID-19 pandemic, a large body of studies using scRNA-seq of PBMCs had revealed detailed changes in gene expression in different immune cell subtypes including different types of T and B cells, NK cells, monocytes, dendritic cells, etc. during and after infection, results from which indicated greatly reduced CD4^+^ and CD8^+^ T-cell numbers and T-cell exhaustion upon SARS-CoV-2 infection. Reduced peripheral mucosal-associated invariable T (MAIT) cell numbers and their migration in and out of the lung had also been observed. Highly activated inflammatory immune responses, including Interferon-gamma (IFN-γ), interleukin-6 (IL-6), and NF-κB responses, had been reported in COVID-19 patients^6–12^. Many studies had revealed immune state differences between people with severe versus mild symptoms, in that strong type I interferon (IFN-α/β) responses were beneficial after COVID-19 infection and attenuated IFN-α/β responses were associated with the development of severe symptoms^13^. In contrast, stronger NF-κB inflammatory responses were associated with more severe symptoms^14^. In addition, increased γδ-T cell and reduced neutrophil contents were reported to be associated with milder symptoms^15^.

Upon SARS-CoV-2 infections, many people developed various degrees of respiratory syndromes, and some with gastrointestinal conditions. It had been reported that blood coagulation disorders, vasculature issues, electrolytes imbalances, renal disorders, metabolic disorders, etc. were major clinical complications with COVID-19^16,17^. The manner in which vaccination would mimic an infection has not been fully evaluated. In this study, we enrolled healthy volunteers who were to be vaccinated with an inactivated SARS-CoV-2 vaccine (Vero Cell)^3^, to participate in antibody and neutralizing antibody testings, as well as detailed clinical laboratory measurements before and at different times after vaccination (two-dose regimens with slightly different schedules were applied). To our surprise, we observed quite consistent pathophysiological changes regarding electrolyte contents, coagulation profiles, renal function as well as cholesterol and glucose metabolic-related features, as if these people had experienced an infection with SARS-CoV-2. In addition, PBMCs scRNA-seq results also indicated consistent reductions in CD8^+^ T cells and increases in monocyte contents, as well as enhanced NF-κB inflammatory signaling, which also mimicked responses after infection. Surprisingly, type I interferon responses, which had been linked to reduced damages after SARS-CoV-2 infection and milder symptoms, appeared to be reduced after vaccination, at least by 28 days post the 1st inoculation. This might suggest that in the short-term (1 month) after vaccination, a person’s immune system is in a non-privileged state, and may require more protection.

## Results

### Longitudinal follow-up of anti-SARS-CoV-2 antibody and neutralizing antibody productions after inoculation of inactivated SARS-CoV-2 vaccine

**Fig. 1:**
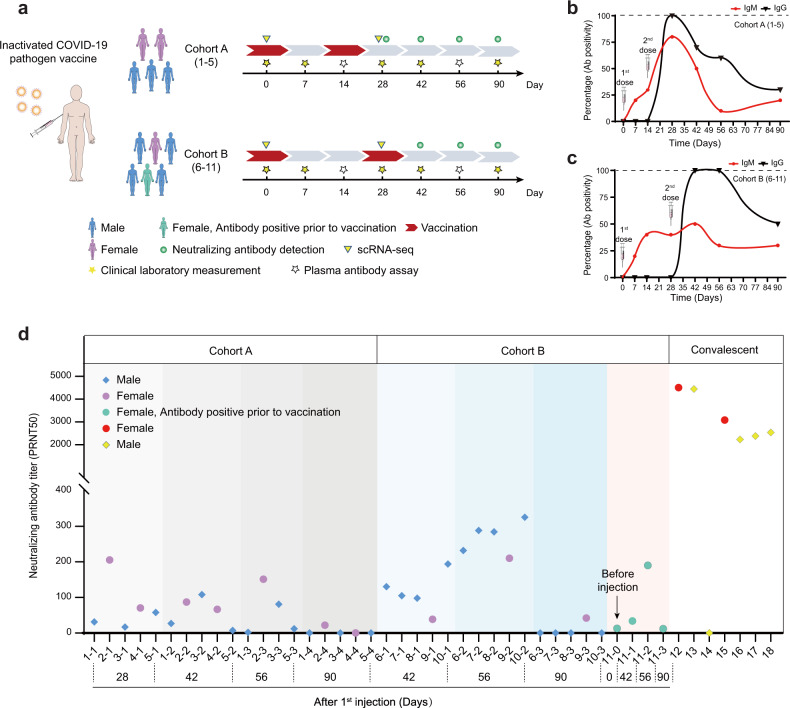
Schematic workflow and SARS-CoV-2 antibody/neutralizing antibody detection after vaccination. **a** Schematic description of vaccine inoculation strategies, blood sample collections, and measurements. **b**, **c** Antibody positivity changes (percent positive/total) over time in cohorts A and B. Volunteers in cohort A were inoculated on days 1 and 14, and in cohort B, on days 1 and 28. Red line represents IgM changes, and black, IgG. **d** Neutralizing antibody titer changes in plasma of volunteers in cohorts A and B after vaccination, as well as those from convalescent individuals tested.

A total of 11 healthy adult volunteers of both sexes, aged 24–47 years, with a BMI of 21.5–30.0 kg/m², were enrolled in this study (Fig. 1a and Supplementary Tables S1 and S2). SARS-CoV-2 vaccine (Vero Cell), inactivated (Beijing Institute of Biological Products Co. Ltd), was administered intramuscularly into the deltoid. Volunteers were divided into two cohorts; five participants (cohort A) were vaccinated with a full dose (4 µg) of inactivated SARS-CoV-2 Vaccine (Vero Cell) on days 1 and 14, and six participants (cohort B) received a full dose of the vaccine on days 1 and 28 (Fig. 1a). One of the volunteers in group B was tested positive for anti-SARS-CoV-2 IgM and IgG right before vaccination, suggestive of potential prior infections. However, there was no record of previous positivity by nucleic acid (NA) diagnosis for COVID-19 (marked green in Fig. 1a). For all follow-up examinations, data from this individual was marked green to track any possible influences from potential prior infections.

Adverse events were monitored daily during the first 7 days after each inoculation and then self-recorded by the participants on diary cards in the following weeks. Overall, adverse reactions were mild (grades 1 or 2) and transient (Supplementary Table S3). Blood samples were collected on days 0, 7, 14, 28, 42, 56, and 90, and urine samples were collected on days 0, 14, 28, 42, and 90. Plasma samples were subjected to anti-SARS-CoV-2 IgM/IgG testing using multiple diagnostic kits, results from the most sensitive kit were used for quantification (Fig. 1b, c). Testing results from cohort A demonstrated that prior to the 2nd inoculation 0% of the participants developed anti-SARS-CoV-2 IgG, but by day 28, which was 2 weeks post the 2nd inoculation, 100% of the participants were tested positive (Fig. 1b). Overall, IgM showed up earlier than IgG, which was expected. IgG and IgM positivity decreased by day 42 and remained at relatively low levels by day 90 in cohort A. For cohort B, no one developed IgG until after 2nd inoculation. Yet by day 42, IgG positivity reached 100% (Fig. 1c) and sustained until day 56, suggesting that the vaccination protocol for cohort B was more efficacious. By day 90, IgG positivity also reduced to 50%, indicating antibody production did not sustain for a long time. We further carried out tests for SARS-CoV-2 neutralizing antibodies^18^ (Fig. 1d), and results also indicated that two inoculations 28 days apart (cohort B) resulted in higher protective antibody titers as compared to two inoculations with 14 days apart (cohort A). On the other hand, it appeared that anti-SARS-CoV-2 neutralizing antibody titers were overall lower than those in COVID-19 convalescent individuals as reported before^3^ (Fig. 1d). By 90 days, neutralizing antibody titers dramatically decreased in all volunteers (Fig. 1d). Interestingly, the individual who was antibody positive prior to vaccination was not more prone to generating neutralizing antibodies as compared to the rest of the participants, suggesting that prior potential infection might not have occurred or may not generate long-lasting protection in the perspective of neutralizing antibody production.

### Alterations in clinical laboratory measurements after vaccination

**Fig. 2:**
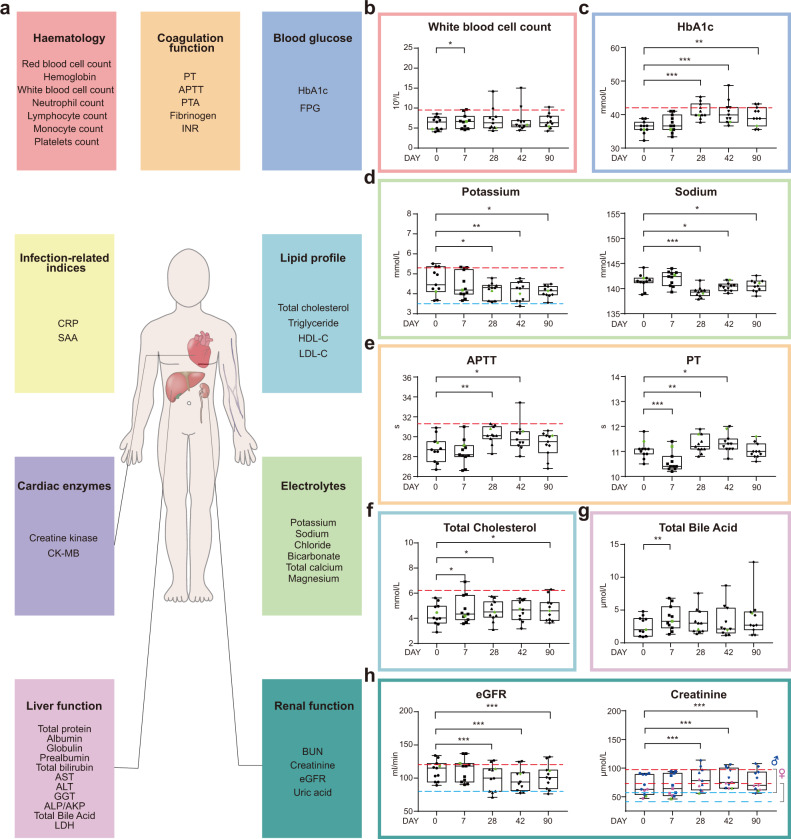
Temporal changes of clinical laboratory measurements after vaccination. **a** Clinical laboratory routine tests include hematologic and coagulation parameters, blood glucose-related and infection-related indices, lipid profile, cardiac enzymes, electrolytes, liver- and renal function-related biomarkers. More information could be found in Supplementary Tables S4 and S5. Laboratory test values of white blood cell count (**b**), HbA1c (**c**), potassium (**d**, left panel), sodium (**d**, right panel), APTT (**e**, left panel), PT (**e**, right panel), total cholesterol (**f**), total bile acid (**g**), eGFR (**h**, left panel), creatinine (**h**, right panel). Data points represent the values of each individual. Box plots showed the 25th, 50th (median), and 75th percentiles. Horizontal dashed lines showed upper normal limits (red) in **b**, **c**, **d** (left panels), **e** (left panel), **f**, **h** and the lower normal limits (blue) in **d** (left panel) and **h**. The *P* values were calculated by the Wilcoxon sign-rank test by comparing the laboratory measurements at each time with the baseline measurements. *P ≤ 0.05, **P ≤ 0.01, ***P ≤ 0.001.

Clinical laboratory routine tests including infection-related indices, hematologic parameters, coagulation function, blood glucose, serum lipids, cardiac function-related enzymes, electrolytes, liver, and renal function-related biomarkers, were measured to reveal safety features of the vaccine (Fig. 2a and Supplementary Tables S4 and S5). White blood cell count was significantly, yet only slightly, increased after vaccination on day 7. No differences were detectable at the following time points (Fig. 2b). To our surprise, quite consistent increases in HbA1c levels were observed in healthy volunteers, regardless of whether they belonged to cohort A or B. By day 28 post the 1st inoculation, three out of 11 individuals reached the prediabetic range (Fig. 2c). By days 42 and 90, medium HbA1c levels appeared to revert back, yet were still significantly higher than those before vaccination. Previous work has demonstrated that diabetic patients with uncontrolled blood glucose levels are more prone to develop severe forms of COVID-19^19^. High blood glucose levels/glycolysis had been shown to promote SARS-CoV-2 replication in human monocytes via the production of mitochondrial reactive oxygen species and activation of HIF1A^20^, therefore presenting a disadvantageous feature.

Serum potassium levels decreased significantly by days 28, 42, and 90 post the 1st inoculation, with one sample below the lower normal limit at day 42 (Fig. 2d, left panel). Similarly, serum sodium levels also decreased following vaccination (Fig. 2d, right panel), indicative of vaccine influences on electrolyte balance. Again, electrolyte imbalance has also been linked to COVID-19^21^. Coagulopathy is another COVID-19-induced clinical condition^22^. We found that coagulation profiles changed significantly after vaccination, in the short-term (7 days) after the 1st inoculation, coagulation profiles were leaning toward shorter Prothrombin Time (PT), whereas the long-term (28 and 42 days) effect was toward activated partial thromboplastin time (APTT) and PT prolongation (Fig. 2e). By day 90, the profiles returned back to those before vaccination (Fig. 2e). Moreover, we found elevated blood cholesterol levels at days 7, 28 after the 1st inoculation, and elevated total bile acid levels were also detected at day 7 (Fig. 2f, g). Renal dysfunction is another clinical condition linked to COVID-19, and by 28, 42, and 90 days after the first inoculation, serum creatinine levels were significantly higher than those before vaccination, resulting in reduced eGFR (Fig. 2h). Most of these clinical features have been reported to be associated with the development of severe symptoms in COVID-19 patients (Supplementary Table S6). Overall, there were no statistically significant differences between cohorts A and B, except for only a few indices (Supplementary Table S7), therefore data from two cohorts were pooled for clinical data presentation and subsequent analyses.

### scRNA-seq revealed dramatic alterations in gene expression of almost all immune cells after vaccination

**Fig. 3:**
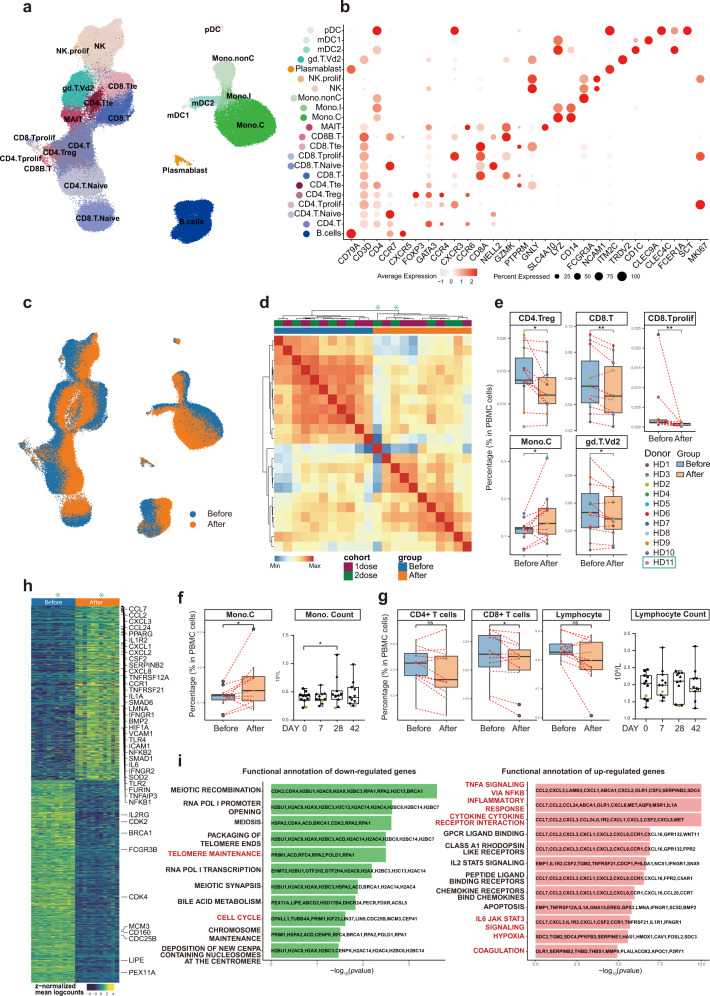
Changes in peripheral immune cell type and subtype compositions as well as gene expression before and 28 days after the 1st inoculation. **a** Cell-type UMAP representation of all merged samples. In total, 22 cell types were identified by cell-type-specific gene expression signatures. In total, 188,886 cells were depicted. **b** Dot plot for cell-type-specific signature genes. Color scale indicated expression levels and point size represented the percentage of cells per cluster/subtype expressing the corresponding gene. **c** UMAP representation representing cells before (blue) and after (orange) vaccination. **d** Heatmap of correlation amongst pseudo-bulk samples. **e** Percentages of specific immune cell subtypes in total PBMCs from each individual before and after vaccination. Box plot depicted sample distribution. Blue boxes represented samples before, and orange, after vaccination. *P* values were based on the Wilcoxon test for comparisons between groups before and after vaccination. **f** Box plots showed changes before and after vaccination in monocyte content from scRNA-seq data (left panel) and clinical laboratory measures (right panel). **g** Box plots showed changes in CD4^+^, CD8^+^ T-cell contents as well as lymphocyte (T + B + NK) contents before and after vaccination from scRNA-seq data (left 3 panels) and laboratory tests (right panel). **h** DEGs identified by pseudo-bulk samples before and after vaccination. **i** Overrepresentation analysis of HALLMARK gene sets from MSigDB demonstrating different immunological features before and after vaccination.

To explore the immunological features of healthy volunteers following vaccination, we performed droplet-based scRNA-seq (10× Genomics) to study transcriptomic profiles of PBMCs from volunteers belonging to either cohort A or B, before and 28 days after vaccination (Fig. 3a and Supplementary Fig. S1a). After preprocessing and low-quality cell elimination (see “Materials and methods”), we obtained 188,886 cells from all PBMC samples, among which 86,685 cells were from cohort A and 102,201 cells from cohort B. All qualified cells were integrated into the unified dataset and subjected to downstream analyses.

Using graph-based clustering of uniform manifold approximation and projection (UMAP)^23^, Single-cell Recognition of cell types (SingleR) algorithm^24^, and manual annotation based on canonical gene markers, we identified 22 cell types or subtypes and performed differential expression analysis amongst all cell types (Fig. 3b and Supplementary Table S8). Cells (cell transcriptomes) from samples before (blue) and after (orange) vaccination were distinctly separated in the UMAP representation for both cohorts, which meant immunological features had changed quite drastically in almost all immune cell types detected, and consistently in all volunteers (Fig. 3c). Among the 11 pairs (before and after) of PBMC samples, 10 pairs were sequenced together and one pair was sequenced separately in a different batch. UMAP distributions were drastically similar regardless of the different batches, suggesting minimal sequencing batch effects (Supplementary Fig. S1b). Two independent batches of sequencing revealed similar changes before and after vaccination, suggesting the changes are real, whereas using the batch effect correction method (Harmony^25^) (Supplementary Fig. S1c–e) would result in over filtration and elimination of the real changes caused by vaccination. Moreover, sample clustering based on the Pearson Correlation coefficient of the transcriptomes indicated that samples from the two cohorts (A and B) intermingled well with each other both before and after vaccination, whereas vaccination-induced changes could clearly be observed (Fig. 3d). Therefore, to increase the statistical power, we combined the two cohorts for subsequent analyses.

To reveal differences in cell-type compositions before and after vaccination, we calculated relative percentages of all cell types in PBMCs of each individual on the basis of scRNA-seq data (Fig. 3e). We observed decreases in contents of CD4^+^ regulatory T cells (CD4.Treg), CD8^+^ T cells (CD8.T), and proliferating CD8^+^ cells (CD8.Tprolif) after vaccination (Fig. 3e). Decreases in γδ-T cell (gd.T.Vd2) contents were also significant (Fig. 3e). In contrast, vaccination increased CD14^+^ classical monocyte (Mono.C) contents (Fig. 3e), consistent with clinical laboratory measurements (Fig. 3f). The overall lymphocyte contents, which included all CD4^+^ T cells, all CD8^+^ T cells, B cells, and NK cells, did not change significantly before and after vaccination, which was also confirmed by clinical laboratory measurements (Fig. 3g). We collected a published dataset from 196 COVID-19-infected patients and controls^7^, and analyzed our data together with that dataset. The result indicated that vaccination-induced changes in cell contents of all five different immune cell subtypes also changed in the same directions in COVID-19 patients as compared to controls, except for proliferating CD8^+^ T cells (Supplementary Fig. S2).

To study detailed gene expression changes induced by vaccination, we merged individual samples into pseudo-bulk samples and used paired sample test to identify differentially expressed genes (DEGs) (Fig. 3h and Supplementary Table S9). Significantly upregulated genes were involved in “TNFα signaling via NF-κB”, “inflammatory responses”, and “cytokine-cytokine receptor interaction”, “IL6-JAK STAT3 signaling”, “coagulation”, “hypoxia”, which had been reported for COVID-19, while cell cycle-related pathways were downregulated (Fig. 3i). These results supported the notion that vaccination mimicked an infection^6–12^.

### Featured immune cell subtype-specific gene expression changes mirrored clinical laboratory alterations

**Fig. 4:**
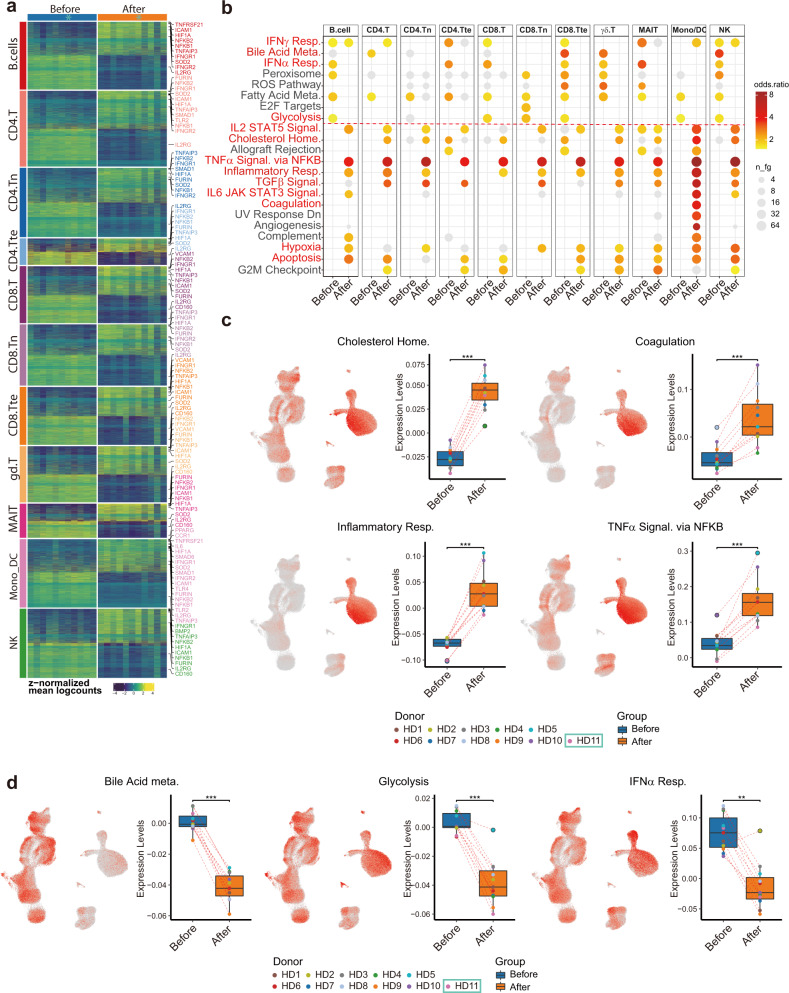
Subtype-specific differential gene expression and gene set overrepresentation analyses depicting common gene expression changes amongst different types of immune cells after vaccination. **a** 11 major immune cell-type-specific DEGs identified by pseudo-bulk data produced by combinations of samples before and after vaccination. Genes with logFC > 0.5 and adjust *P* < 0.05 were included. **b** Overrepresentation analysis of HALLMARK gene sets from MSigDB amongst 11 major cell types demonstrated common changes in gene sets representing altered immunological states before and after vaccination. **c**, **d** UMAP visualization colored by average expression scores (levels) based on differential enrichment pathway. Box plot depicting the expression score distribution before and after vaccination.

Prior to the elucidation of the functional heterogeneity and cell-type-specific gene expression changes between samples before and after vaccination, we grouped cells into 11 major types: (1) naive-state CD4^+^ T cells, (2) naive-state CD8^+^ T cells, (3) CD4^+^ helper T cells (including CD4.T, CD4.Treg, and CD4.Tprolif), (4) CD8^+^ cytotoxic T cells (including CD8.T, CD8B.T, and CD8.Tprolif), (5) MAIT, (6) γδ-T cells, (7) NK cells (including NK, NK proliferative), (8) B/plasmablast cells (including B cells and plasmablasts), (9) monocytes/dendritic cells (including classical mono, intermediate mono, non-classical mono, myeloid DC1, myeloid DC2, and plasmacytoid DC), (10) CD4^+^ terminal effector T cells, and (11) CD8^+^ terminal effector T cells. Following eleven major cell-type categorizations, we performed sample-level comparisons by aggregating gene expression across major cell types within each donor and then performed differential expression analysis using muscat^26^. We identified diferentially expressed genes (DEGs) among all major cell types (Fig. 4a and Supplementary Table S10) and conducted gene functional analysis (Fig. 4b). Echoing the clinical measurement results, genes related to “cholesterol homeostasis”, “coagulation”, and “inflammatory response” (CXCL8, CD14, IL6, and TNFRSF1B), “TNFα signaling via NF-κB” (NFKB1, NFKB2, NFKBIE, TNFAIP3, and TNFSF9) and “hypoxia” (HIF1A) were upregulated. In addition, “TGFβ signaling”, “IL2-STAT5 signaling” (IFNGR1, MAPKAPK2, and CASP3), and “IL6-JAK-STAT3 signaling”-related genes were also upregulated (Fig. 4c). To visualize which cell types were enriched for those signatures, we performed gene module scoring and displayed the scores on UMAP coordinates as well as grouped box plots (Fig. 4c and Supplementary Table S11). Interestingly, “inflammatory response” genes were highly expressed in monocytes and after vaccination further increased (Fig. 4c), suggesting monocytes were one of the major cell types participating in inflammatory responses after vaccination. In contrast, genes related to “glycolysis”, “bile acid metabolism”, and “type I interferon (IFN-α/β) response” were downregulated, consistent with our clinical data and the pathophysiology of COVID-19^13^ (Fig. 4d).

### Most common changes in multiple immune cell subtypes revealed increases in NF-κB signaling and decreases in IFN-α/β responses

**Fig. 5:**
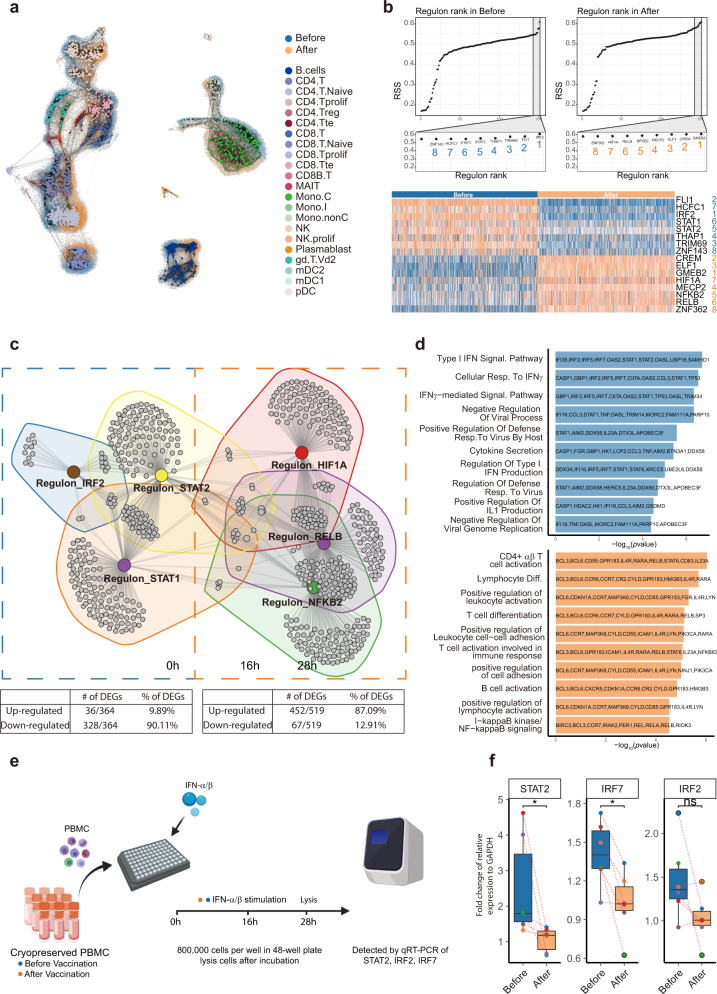
Identification of master regulons and their regulatory networks before and after vaccination. **a** Visualization for the “similarity-structure-associating” metacells on the original scRNA-seq data. Metacells were color-coded according to their cell-type annotations. The original scRNA-seq data were color-coded “blue” and “orange” to represent samples “before” and “after” vaccination, respectively. **b** Top panels: rank of regulons in samples before (left) and after (right) vaccination, based on Regulon Specificity Score (RSS). Bottom panels: heatmap of top-ranked regulon activities before (blue) and after (orange) vaccination based on AUCell scores. Names of the regulons are color (blue/orange) and number coded (1–8). **c** Network of regulons and their target genes. The table below indicated the proportion of genes within the regulons which were up- or downregulated after vaccination. **d** Gene functional annotation and related genes before (blue) and after (orange) vaccination. **e** Schematic overview of the experiment. **f** After treatment with IFN-α/β, PBMCs from volunteers after vaccination had reduced expression of genes associated with type I interferon responses as compared to those before vaccination. Paired Wilcoxon test was used. *P ≤ 0.05, *n* = 6.

Given that clusters of genes changed their expression dramatically among all major cell types, we hypothesized that there might be some transcription factors serving as master regulators leading to immunological alterations. To solve the computational challenges associated with such a big dataset, we used the MetaCell algorithm^27^ to aggregate homogeneous groups of cells into metacells, and finally produced 1857 metacells (893 before and 964 after vaccination) to represent the whole structure of the scRNA-seq data (Fig. 5a). Those metacells were then applied to “single-cell regulatory network inference and clustering (SCENIC)”^28,29^ to construct the gene regulatory networks. The workflow produced a list of 157 “regulons”, which included transcription factors and their direct targets. Regulon activities were scored using AUCell to access averaged enrichment of all genes belonging to each regulon in each metacell, as well as averaged regulon gene enrichment in all 893 metacells before vaccination, and 964 metacells after vaccination. Top-ranked (most active) eight regulons upregulated and eight regulons downregulated after vaccination were identified (Fig. 5b). We selected 3 + 3 typical regulons to construct a regulatory network as presented in Fig. 5c (Supplementary Table S12). The network showed two distinct groups, one is consisted of IRF2, STAT1 and STAT2, which were downregulated after vaccination, and the other, contained RELB, NFKB2, and HIF1A, which were upregulated after inoculation. The GO terms of the upregulated network are predominantly related to lymphocyte differentiation, activation, and “Germinal Center Formation”, which suggested that T cells and B cells were activated after vaccination. In addition, NF-κB signaling was also elevated after vaccination. The downregulated network was enriched for many interferons-related pathways and Cytokine Secretion (Fig. 5d and Supplementary Table S13). This suggested that vaccination might inhibit interferon responses in the peripheral immune system, by reducing the activities of regulons STAT1, STAT2, and IRF2, which were thought to be master transcription factors driving type I and III interferon signaling^30,31^.

To confirm vaccination-induced inhibition of interferon responses revealed by scRNA-seq, we stimulated PBMCs from vaccinated individuals before and 28 days after vaccination with IFN-α/β. After 16 h of culturing and 12 h of stimulation, we used RT-qPCR to measure the relative expression of master regulators IRF2, IRF7, and STAT2. STAT2 and IRF7 were significantly downregulated after vaccination, yet IRF2 showed a trend of downregulation (Fig. 5e, f). The regulon analyses indicated that the states of the peripheral immune system after vaccination had reduced type I interferon responses, indicative of attenuated general antiviral abilities at least 28 days after the first inoculation.

### Vaccination-induced inflammatory responses in monocytes

**Fig. 6:**
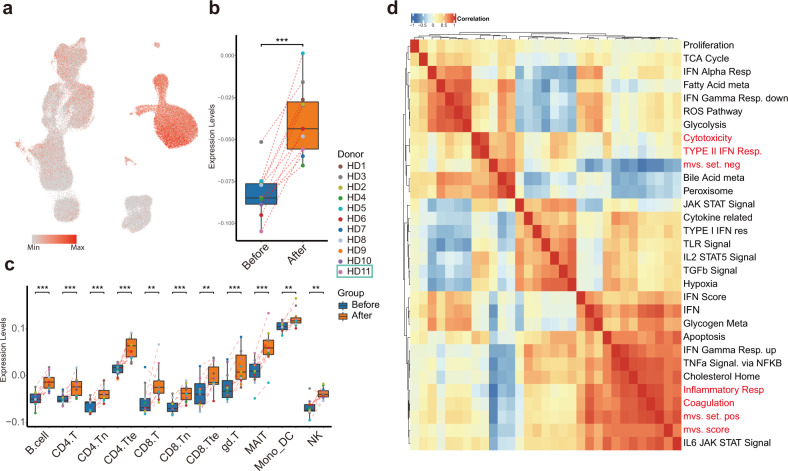
Monocytes displayed high MVS scores and MVS score-correlated pathways. **a** UMAP visualization colored by MVS scores. **b** MVS scores in pseudo-bulk samples combining all cell types showed upregulation after vaccination. **c** Box plots depicting the score distribution amongst 11 major immune cell types before and after vaccination. **d** Heatmap of correlation between MVS scores and enriched pathways of differentially expressed genes before and after vaccination.

Recent reports have described conserved host immune response signatures to respiratory viral infections, namely the Meta-Virus Signature (MVS), which is also conserved in SARS-CoV-2 infection^32,33^. Higher MVS scores are associated with infection^32,33^. In all, 380 (158 positively- and 222 negatively contributed to MVS scores) out of 396 (161 positively- and 235 negatively contributed) genes selected for MVS measurement were detected in our dataset. To investigate host immune responses after vaccination with inactivated SARS-CoV-2, we separated the positive and negative gene sets and calculated MVS scores (Fig. 6a). The MVS scores were substantially higher after vaccination (Fig. 6b, c), suggesting that vaccination mimicked an infection. Interestingly, the positive MVS gene set was predominantly expressed in monocytes, while the negative set in lymphocytes, indicating different cell-type-specific immune responses would take place after vaccination (Supplementary Fig. S3a, b).

To investigate which pathways were associated with MVS-positive gene set and MVS-negative gene set, we calculated Spearman correlation among MVS gene sets scores and previously identified differentially enriched pathways using our scRNA-seq data (Fig. 6d). The most highly correlated pathway with MVS score and MVS-positive set was “Inflammatory response signaling”, which was strikingly upregulated in monocyte after vaccination, together with CD14, FPR1, C5AR1, NAMPT, NLRP3, CDKN1A, and IFNGR2. Whereas, MVS-negative set correlated well with “Cytotoxicity signature”, represented by NKG7, CCL4, CST7, PRF1, GZMA, GZMB, IFNG, and CCL3 expression, significantly decreased in many T-cell subtypes but not NK cells after vaccination (Supplementary Fig. S3c).

## Discussion

This is a comprehensive investigation of the pathophysiological changes, including detailed immunological alterations in people after COVID-19 vaccination. Results indicated that vaccination, in addition to stimulating the generation of neutralizing antibodies, also influenced various health indicators including those related to diabetes, renal dysfunction, cholesterol metabolism, coagulation problems, electrolyte imbalance, in a way as if the volunteers experienced an infection. scRNA-seq of PBMCs from volunteers before and after vaccination revealed dramatic changes in immune cell gene expression, not only echoing some of the clinical laboratory measures but also suggestive of increased NF-κB-related inflammatory responses, which turned out to be mainly taking place in classical monocytes. Vaccination also increased classical monocyte contents. Moreover, the gene set positively contributing to MVS scores, also known to be associated with severe symptom development, was highly expressed in monocytes. Type I interferon (IFN-α/β) responses, supposedly beneficial against COVID-19, were downregulated after vaccination. In addition, the negative MVS genes were highly expressed in lymphocytes (T, B, and NK cells), yet showed reduced expression after vaccination. Together, these data suggested that after vaccination, at least by day 28, other than generation of neutralizing antibodies, people’s immune systems, including those of lymphocytes and monocytes, were perhaps in a more vulnerable state.

Interestingly, our preliminary data demonstrated that if we pre-incubated RBD of SARS-CoV-2 with the PBMCs (from volunteers before and after vaccination) and then treated the cells with IFN-α/β, type I interferon responses were actually enhanced in PBMCs after vaccination, suggesting that perhaps vaccination, while reduced a person’s general antiviral ability, enhanced adaptive immune function specifically towards SARS-CoV-2 (Supplementary Fig. S4a). On the other hand, comparing PBMCs before vaccination, pre-treatment of SARS-CoV-2 S-RBD appeared to reduce type I interferon responses (*P* < 0.05, IRF2, IRF7, STAT2) (Supplementary Fig. S4b), suggesting 1st time exposure of the viral peptide would actually cause a reduction in type I interferon responses in PBMC. These in vitro data nicely supported the scRNA-seq results.

It is worth mentioning that one individual in cohort A who was on antibiotics, happened to not having reduced gene expression linked to type I interferon responses, and this individual also had the highest neutralizing antibody titer within the cohort. We further calculated Pearson’s Correlation Coefficient between neutralizing antibody titers and inflammatory responses measured by averaged gene expression of genes associated with TNFα Signaling via NF-κB and interferon-α (type I interferon) responses. The results were 0.32 and 0.39 with *P* > 0.05 (Supplementary Fig. S4c), respectively, suggesting immune response changes and adaptive immune protection of the vaccine do not appear to be highly correlated. Whether antibiotics may influence vaccine efficacy remains to be determined. It is also rather interesting that while cohorts A and B had different anti-SARS-CoV-2 antibody production profiles, their PBMCs scRNA-seq results were drastically similar, including their B-cell scRNA-seq data (Supplementary Fig. S5a–c). It should be noted that after vaccination, the majority of responsive B cells, particularly those producing mature anti-COVID-19 antibodies (IgG) including memory B cells, should be primarily located in peripheral lymphatic tissues such as lymph nodes and the spleen, while only a few mature B cells would exist in the circulation. Therefore, the B-cell population in PBMCs preparations may not reflect the whole spectrum of humoral immunity.

The analyses presented in this study, particularly, scRNA-seq of PBMCs had not been performed for previous vaccine evaluations, whether the changes in immune system function-related genes were COVID-19-specific or could be generally applied to other vaccines or other types of COVID-19 vaccines remained to be determined. However, these types of detailed analyses should be overall beneficial to vaccine development and applications. Our study postulates that it is imperative to consider the potential long-term impact of vaccination to certain medical conditions^34^ or to general human health.

## Materials and methods

### Participants, clinical data collection, and procedures

Healthy adult volunteers were recruited to the program. All subjects underwent a physical examination and completed a questionnaire by trained doctors. Healthy adult aged 18–60 years, with axillary temperature ≤ 37.0 °C, negative for SARS-CoV-2 nucleic acid test, and willing to complete all scheduled study processes were enrolled in the study. People with epilepsy, brain or mental diseases, history of allergies, uncontrolled major chronic illnesses, and clinically significant abnormal findings on biochemistry, hematology tests were excluded. Pregnant or breastfeeding women were also excluded. This study was approved by the Ethics Committee of Shanghai East Hospital in accordance with the principles of the Helsinki Declaration (No.2020 (096)). Written informed consents were obtained from all participants before enrollment.

A total of 11 participants were enrolled and vaccinated to evaluate the clinical safety and dynamic changes in the immune system. Among these, five participants (cohort A) were vaccinated with 4 μg dose of inactivated SARS-CoV-2 Vaccine (Vero Cell) on days 1 and 14, and six participants (cohort B) received a 4 μg dose of the vaccine on days 1 and 28. Inactivated SARS-CoV-2 Vaccine (Vero Cell) (China Biotechnology Group Corporation) was administered intramuscularly into the deltoid. All vaccines were approved by the National Institutes for Food and Drug Control of China.

Laboratory safety tests including infection-related indices (C-reactive protein, serum amyloid A protein), hematologic parameters (white blood cell counts, neutrophil counts, lymphocyte counts, monocyte counts, red blood cell counts, hemoglobin, platelet counts), coagulation function-related indices (prothrombin time, activated partial thromboplastin time/APTT, fibrinogen, prothrombin activity/PT, international normalized ratio/INR), blood glucose-related parameters (fasting plasma glucose, HbA1c), serum lipid (total cholesterol, triglyceride, HDL-C, LDL-C), cardiac function-related enzymes (creatinine kinase, CK-MB), electrolytes (potassium, sodium, chloride, bicarbonate, total calcium, magnesium), liver function-related biomarkers (e.g., albumin, alanine aminotransferase/ALT, aspartate aminotransferase/AST, total bilirubin, and etc.), renal function-related markers (creatinine, uric acid, blood urine nitrogen/BUN, estimated glomerular filtration rate/eGFR) were measured.

### COVID-19 antibody (IgG/IgM) testing

A number of commercially available COVID-19 antibody (IgG/IgM) rapid testing kits including “Innovita (S protein specific)”, “GenBody (N protein specific)”, “Livzon (S + N proteins)”, and “AbKhan (S + N proteins)” were used to test anti-COVID-19 (IgM/IgG) positivities of plasma from volunteers before and at different times after vaccination. The “AbKhan” kit was most sensitive and data were used in this study.

### Neutralizing antibody test by PRNT

Serum samples were each tested using a plaque reduction neutralization test (PRNT) assay for SARS-CoV-2 (2019-nCoV-WIV04) in the BSL-3 laboratory. Briefly, sera were heat-inactivated at 56 °C for 30 min and diluted to 1:50, followed by threefold serial dilutions (1:50, 1:150, 1:450, 1:1350, 1:4050, and 1:12,150). Sera were then mixed with 100 PFU of virus and incubated at 37 °C for 1 h. The virus–serum dilution mixtures and virus control were then inoculated into Vero E6 cell monolayers in 24-well plates for 1 h before adding an overlay medium including 1.5% methylcellulose at 37 °C for 4–5 days to allow plaque development. Then the plates were fixed and stained with 2% crystal violet in 30% methanol for 30 min at room temperature, and the plaques were manually counted and measured. The PRNT titer was calculated based on a 50% reduction in plaque count (PRNT50).

### Preparation of single-cell suspensions, single-cell RNA library preparation, and sequencing

The PBMCs were isolated from heparinized venous blood from healthy volunteers using a Ficoll-Paque^TM^ PLUS Media (GE Healthcare Inc.) according to the standard density-gradient centrifugation method provided by the manufacturer. PBMCs were frozen in freezing media (70% RPMI-1640, 20% FBS, and 10% DMSO), and stored in liquid nitrogen until use. Single-cell capture and library construction were performed using the Chromium Single Cell 5′ Library & Gel Bead kit (10× Genomics) according to the manufacturer’s instructions. Libraries were sequenced using the Novaseq 6000 platform (Illumina).

### scRNA-seq data analysis and statistics

Single-cell sequencing data were aligned and quantified using kallisto/bustools (KB, v0.25.0)^35^ against the GRCh38 human reference genome downloaded from the 10× Genomics official website. Preliminary counts were then used for downstream analyses. We made a pipeline to process data. Briefly, cells with less than 200 genes were filtered out, the logarithmic normalized counts and top 3000 highly variable genes (HVGs) selection were performed by Scanpy^36^.

We excluded specific genes from HVGs including mitochondrial genes, immunoglobulin genes, and genes linked to poorly supported transcriptional models (annotated with the prefix “Rp-”). Then principal component analysis (PCA) was performed utilizing the HVGs and Harmony algorithm was used to remove batch effects^25^. We used the PARC approach to identify clusters^37^ and selected features by “FeatureSelectionByEnrichment” function from cytograph2 algorithm^38^, followed by another round of PCA, Harmony, and PARC. Subsequently, we calculated K nearest neighbors in a KNN graph, performed uniform manifold approximation and projection (UMAP) by Pegasuspy^39^, and identified clusters by PARC. In addition, we applied Scrublet^40^ to identify potential doublets.

Quality control was applied to clusters based on output of the first round of the pipeline:

1. Clusters with more than 20% cells of which doublet score > 0.4 were defined as doublets clusters.
2. Clusters with more than 20% cells that had > 20% of their transcripts mapped to mitochondrial genes were defined as low-quality clusters.
3. Clusters with more than 20% cells that had < 0.05% of their transcripts mapped to mitochondrial genes were defined as nuclei.
4. Median expression of PPBP, PF4, HBB, HBA2 > 0, indicating erythrocytes and platelets.
5. Less than 50 cells.
6. Detected gene numbers < 1000.
7. Ratio of mean of total UMIs and mean of detected genes < 2.
8. Scrublet identified doublets.
9. Using DBSCAN^41^ to remove outliers.

After removing low-quality cells, we annotated cells by single-cell recognition of cell types (SingleR) algorithm, referring to Monaco immune datasets^42^.

Qualified cells were subjected to downstream analysis. Similarly, we rerun the pipeline to identify main cell types including T cells (CD3D, CD3E, CD3G, CD40LG, CD8A, CD8B), B cells (MS4A1, CD79A, CD79B), NK cells (GNLY, NKG7, TYROBP, NCAM1), and monocytes (CST3, LYZ). In addition, we run the pipeline on each type of cells, respectively, and further identified subtypes based on the SingleR-identified cell types and well-characterized markers (Fig. 3b).

### Comparing immune cell proportion

For samples from PBMCs, we calculated immune cell proportions for each major cell type and underlying subtypes. For each sample, the cell-type proportion was calculated by the number of cells in a certain cell type divided by the total number of cells. To identify changes in cell proportions between samples in different groups, we performed a Wilcoxon test on the proportions of each major cell types as well as cell subtypes across different groups (Supplementary Fig. S2). Only those cell types with statistically significant differences (*P* < 0.05) in proportions are shown in Fig. 3e.

### Differential expression analysis, gene sets overrepresentation analysis, and score signature modules

To investigate immunological feature alterations, we identified DEGs by muscat algorithm^26^ with default parameters. Briefly, we first sum-collapsed the data, summing UMIs across cells for each healthy donor, to produce a bulk RNA-seq style UMIs profile for each sample. Afterward, the aggregated counts were loaded onto pbDS function to identify DEGs, and heatmaps were plotted by pbHeatmap function. Gene set overrepresentation analysis of DEGs (logFC > 0.5 and adjusted *P* < 0.05) were performed using one-sided Fisher’s exact test (as implemented in the “gsfisher” R package) with “HALLMARK”, “KEGG”, and “REACTOME” gene sets derived from MSigDB. Gene sets with *P* < 0.05 were considered to be significant. Signature module scores were calculated via “AddModuleScore” function, with default settings in Seurat. Briefly, for each cell, the score was defined as the average expression of the signature gene list subtracting the average expression of the corresponding control gene list^43^. Gene lists used for analysis are provided in Supplementary Table S11.

### Metacell analysis

We used the R package “MetaCell”^27^ to analyze the data. We removed specific mitochondrial genes, immunoglobulin genes, and genes linked to poorly supported transcriptional models (annotated with the prefix “Rp-”). We then filtered cells with less than 500 UMIs. Gene features were selected using the parameter Tvm = 0.08 and a minimum total UMI count > 100. We subsequently performed hierarchical clustering of the correlation matrix between those genes (filtering genes with low coverage and computing correlation using a down-sampled UMI matrix) and selected gene clusters containing anchor genes. We used *K* = 100, and 500 bootstrap iterations and otherwise standard parameters. Metacells were annotated by the most abundant cell types composing each metacell.

### Gene regulatory network analysis

For identification and scoring of regulon activity, we employed pySCENIC^28,29^ workflow on log-normalized metacells data to determine sets of co-expressed genes. We linked direct targets to their corresponding transcription factors using RcisTarget databases (v1.2.1), and retained putative downstream genes with enriched DNA motifs at 10 kb or 500 bp from the transcription start site (normalized enrichment score > 3). Finally, we used AUCell function to score activity of each regulon across cells in the dataset, which was computed as the sum of genes expressed per regulon and produced binary activity matrices based on cutoffs manually adjusted after inspecting the distributions of AUC scores. Regulon specificity scores (RSS) were calculated by the “regulon_specificity_scores” function from pySCENIC algorithm with default parameters.

### Analysis of IFN-α/β response of PBMCs

PBMCs were isolated from heparinized blood by Ficoll-Hypaque at 400× *g* for 30 min. The PBMCs (1 × 10^6^ ml^−1^) of donors before and after vaccination were then seeded in 48-well culture plates with RPMI-1640 containing 5% knockout serum replacement and 0.032% heparin. The next day, medium was exchanged and cells were treated with 100 ng/ml IFN-α and 10 ng/ml IFN-β for 12 h. Some cells were pre-treated with 250 ng/ml RBD for 16 h, followed by IFN-α/β treatment for 12 h. Following washing and extraction of total RNA, real-time quantitative PCR was performed to detect the expression of type I interferon response-associated genes. Fold changes relative to GAPDH were calculated by 2^-ΔΔCt^ and expressed as means ± SEM. Differences between groups were evaluated using paired Student’s *t*-test and considered significant when *P* < 0.05.

### Statistical analysis

Clinical data were summarized using mean (standard deviation), median (Q1, Q3), or number (percentage), when appropriate. The Wilcoxon signed-rank test was used to compare paired medians over time for laboratory characteristics. In addition, Wilcoxon sum-rank test was used to compare the median changes from baseline between cohorts A and B. We graded adverse events according to the scale issued by the China National Medical Products Administration (https://www.nmpa.gov.cn/xxgk/ggtg/qtggtg/20191231111901460.html) and the judgment of laboratory test results was based on the reference value range of the local population. All statistical tests were two-sided. Statistical significance was defined as *P* ≤ 0.05. Statistical analyses were performed using SAS v9.4 (SAS Institute Inc., Cary, NC, USA).

## Supplementary information

Supplementary Figures

Supplementary_Tables_Summary

## Data Availability

The accession numbers for the sequencing raw data and processed data in this paper are Genome Sequence Archive in BIG Data Center (GSA, Beijing Institute of Genomics, Chinese Academy of Sciences): HRA001150.

## References

[1] Mulligan MJ (2020). Phase I/II study of COVID-19 RNA vaccine BNT162b1 in adults. Nature.

[2] Jackson LA (2020). An mRNA vaccine against SARS-CoV-2—preliminary report. N. Engl. J. Med..

[3] Xia S (2021). Safety and immunogenicity of an inactivated SARS-CoV-2 vaccine, BBIBP-CorV: a randomised, double-blind, placebo-controlled, phase 1/2 trial. Lancet Infect. Dis..

[4] Ramasamy MN (2020). Safety and immunogenicity of ChAdOx1 nCoV-19 vaccine administered in a prime-boost regimen in young and old adults (COV002): a single-blind, randomised, controlled, phase 2/3 trial. Lancet.

[5] Xia S (2020). Effect of an inactivated vaccine against SARS-CoV-2 on safety and immunogenicity outcomes: interim analysis of 2 randomized clinical trials. J. Am. Med. Assoc..

[6] Meckiff BJ (2020). Imbalance of regulatory and cytotoxic SARS-CoV-2-reactive CD4(+) T cells in COVID-19. Cell.

[7] Ren, X. et al. COVID-19 immune features revealed by a large-scale single-cell transcriptome atlas. *Cell*10.1016/j.cell.2021.01.053 (2021). 10.1016/j.cell.2021.10.023 PMC8582084 34767776

[8] Bernardes JP (2020). Longitudinal multi-omics analyses identify responses of megakaryocytes, erythroid cells, and plasmablasts as hallmarks of severe COVID-19. Immunity.

[9] Su Y (2020). Multi-Omics resolves a sharp disease-state shift between mild and moderate COVID-19. Cell.

[10] Wen W (2020). Immune cell profiling of COVID-19 patients in the recovery stage by single-cell sequencing. Cell Discov..

[11] Heming M (2021). Neurological manifestations of COVID-19 feature T cell exhaustion and dedifferentiated monocytes in cerebrospinal fluid. Immunity.

[12] Flament H (2021). Outcome of SARS-CoV-2 infection is linked to MAIT cell activation and cytotoxicity. Nat. Immunol..

[13] Hadjadj J (2020). Impaired type I interferon activity and inflammatory responses in severe COVID-19 patients. Science.

[14] Kusnadi, A. et al. Severely ill COVID-19 patients display impaired exhaustion features in SARS-CoV-2-reactive CD8(+) T cells. *Sci. Immunol*. **6**, 10.1126/sciimmunol.abe4782 (2021). 10.1126/sciimmunol.abe4782 PMC8101257 33478949

[15] Carissimo G (2020). Whole blood immunophenotyping uncovers immature neutrophil-to-VD2 T-cell ratio as an early marker for severe COVID-19. Nat. Commun..

[16] Guan WJ (2020). Clinical characteristics of coronavirus disease 2019 in China. N. Engl. J. Med..

[17] Huang C (2020). Clinical features of patients infected with 2019 novel coronavirus in Wuhan, China. Lancet.

[18] Lau EHY (2021). Neutralizing antibody titres in SARS-CoV-2 infections. Nat. Commun..

[19] Lim S, Bae JH, Kwon HS, Nauck MA (2021). COVID-19 and diabetes mellitus: from pathophysiology to clinical management. Nat. Rev. Endocrinol..

[20] Codo AC (2020). Elevated glucose levels favor SARS-CoV-2 Infection and monocyte response through a HIF-1alpha/glycolysis-dependent axis. Cell Metab..

[21] Lippi G, South AM, Henry BM (2020). Electrolyte imbalances in patients with severe coronavirus disease 2019 (COVID-19). Ann. Clin. Biochem..

[22] Zhang Y (2020). Coagulopathy and antiphospholipid antibodies in patients with Covid-19. N. Engl. J. Med..

[23] Becht, E. et al. Dimensionality reduction for visualizing single-cell data using UMAP. *Nat. Biotechnol*. 10.1038/nbt.4314 (2018). 10.1038/nbt.4314 30531897

[24] Aran D (2019). Reference-based analysis of lung single-cell sequencing reveals a transitional profibrotic macrophage. Nat. Immunol..

[25] Korsunsky I (2019). Fast, sensitive and accurate integration of single-cell data with Harmony. Nat. Methods.

[26] Crowell HL (2020). muscat detects subpopulation-specific state transitions from multi-sample multi-condition single-cell transcriptomics data. Nat. Commun..

[27] Baran Y (2019). MetaCell: analysis of single-cell RNA-seq data using K-nn graph partitions. Genome Biol..

[28] Van de Sande B (2020). A scalable SCENIC workflow for single-cell gene regulatory network analysis. Nat. Protoc..

[29] Aibar S (2017). SCENIC: single-cell regulatory network inference and clustering. Nat. Methods.

[30] Mu J (2020). SARS-CoV-2 N protein antagonizes type I interferon signaling by suppressing phosphorylation and nuclear translocation of STAT1 and STAT2. Cell Discov..

[31] Boudewijns R (2020). STAT2 signaling restricts viral dissemination but drives severe pneumonia in SARS-CoV-2 infected hamsters. Nat. Commun..

[32] Andres-Terre M (2015). Integrated, multi-cohort analysis identifies conserved transcriptional signatures across multiple respiratory viruses. Immunity.

[33] Zheng, H. et al. Multi-cohort analysis of host immune response identifies conserved protective and detrimental modules associated with severity across viruses. *Immunity*, 10.1016/j.immuni.2021.03.002 (2021). 10.1016/j.immuni.2021.03.002 PMC7988739 33765435

[34] Hviid A, Hansen JV, Frisch M, Melbye M (2019). Measles, mumps, rubella vaccination and autism. Ann. Intern. Med..

[35] Bray NL, Pimentel H, Melsted P, Pachter L (2016). Near-optimal probabilistic RNA-seq quantification. Nat. Biotechnol..

[36] Wolf FA, Angerer P, Theis FJ (2018). SCANPY: large-scale single-cell gene expression data analysis. Genome Biol..

[37] Stassen SV (2020). PARC: ultrafast and accurate clustering of phenotypic data of millions of single cells. Bioinformatics.

[38] Zeisel A (2018). Molecular architecture of the mouse nervous system. Cell.

[39] Li B (2020). Cumulus provides cloud-based data analysis for large-scale single-cell and single-nucleus RNA-seq. Nat. Methods.

[40] Wolock SL, Lopez R, Klein AM (2019). Scrublet: computational identification of cell doublets in single-cell transcriptomic data. Cell Syst..

[41] Ester M, Kriegel H-P, Sander J, Xu X (1996). A density-based algorithm for discovering clusters in large spatial databases with noise. Kdd.

[42] Monaco G (2019). RNA-Seq signatures normalized by mRNA abundance allow absolute deconvolution of human immune cell types. Cell Rep.

[43] Tirosh I (2016). Dissecting the multicellular ecosystem of metastatic melanoma by single-cell RNA-seq. Science.

