# Supplementary material for: Comprehensive investigations revealed consistent pathophysiological alterations after vaccination with COVID-19 vaccines
Source: Cell Discov. 2021 Oct 26;7:99. doi: 10.1038/s41421-021-00329-3 (PMC8546144; doi:10.1038/s41421-021-00329-3)
Supplement: Supplementary file 1 — Supplementary Figures [file 41421_2021_329_MOESM1_ESM.pdf]

Supplementary Fig. S1

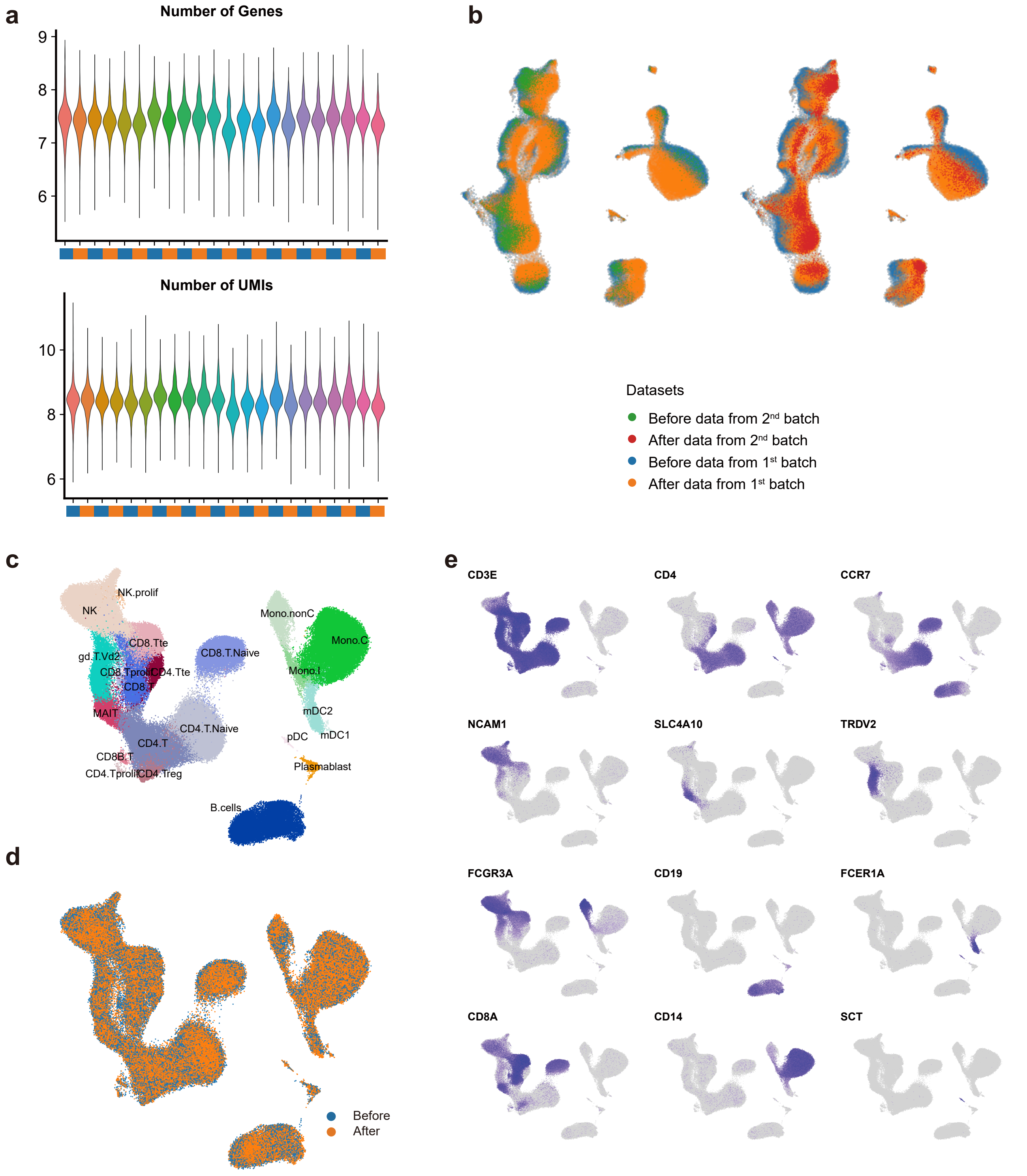

Supplementary Fig. S1 a QC analysis of scRNA-seq data. Numbers of genes, numbers of mRNA molecules (counts) in each sample. **b** UMAP distribution of samples from the 2nd batch of sequencing overlaid on top of data from the 1st batch showing consistent changes before and after vaccination. **c, d, e** UMAP representation of all cells after batch effect correction eliminated changes before and after vaccination (**d**), but clearly demonstrated cell-type specific expression of marker genes (**e**).

Supplementary Fig. S2

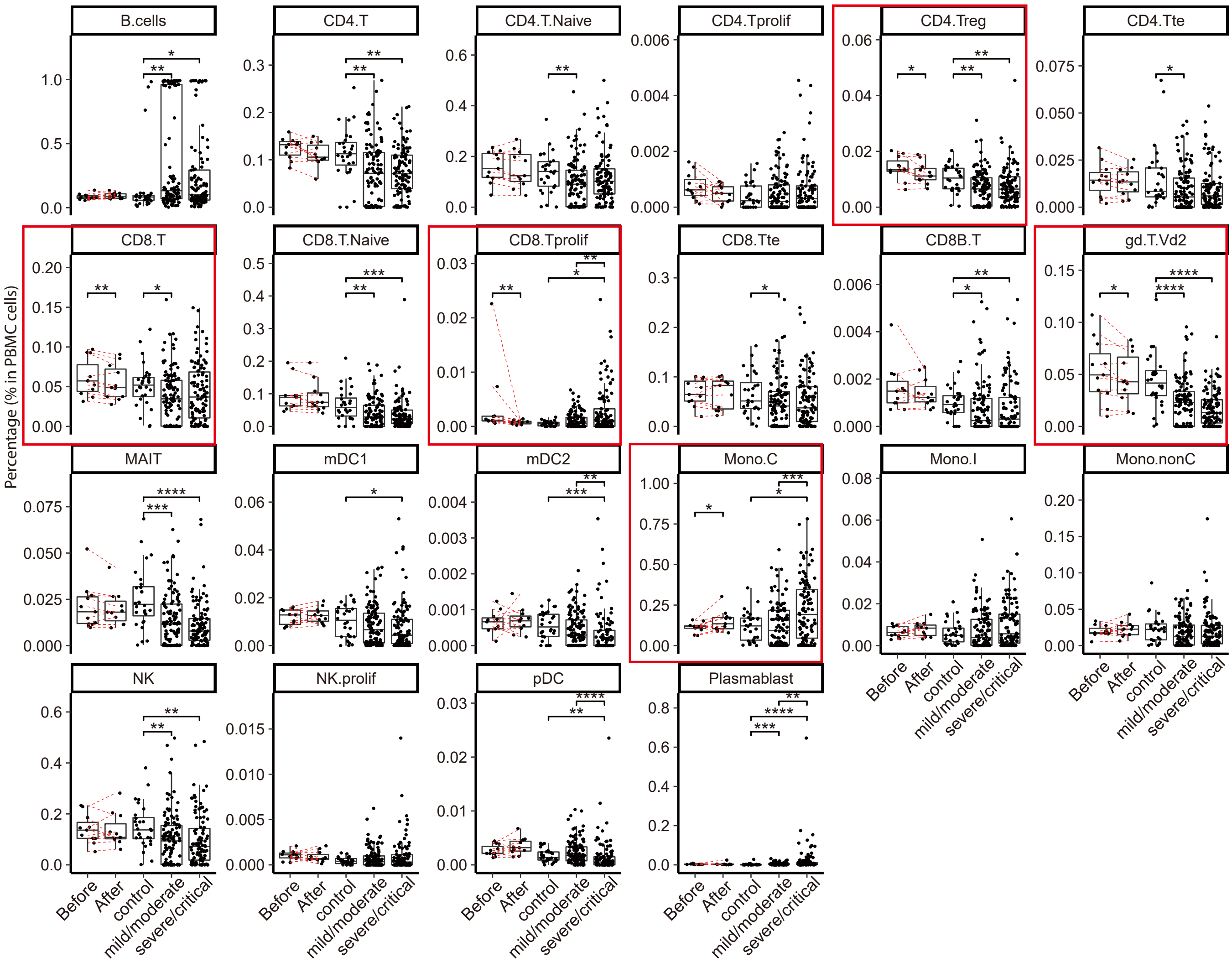

Supplementary Fig. S2 Cell type specific content changes before and after vaccination, in relate to changes in mild and severe COVID-19 patients and healthy individuals.

Supplementary Fig. S3

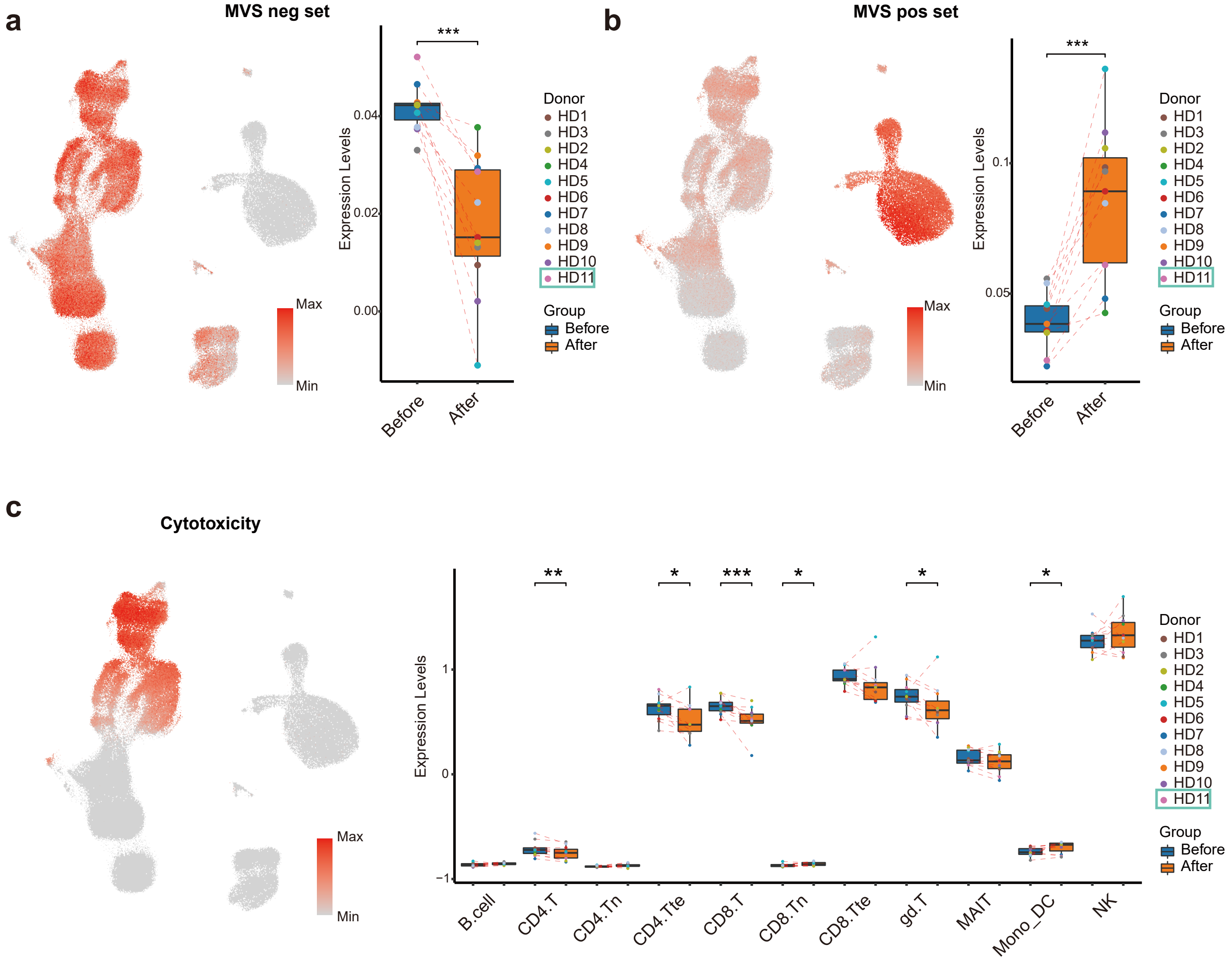

Supplementary Fig. S3 a Averaged expression of genes negatively contributing to MVS scores on UMAP plot and box plots demonstrating down-regulation of this gene set after vaccination. **b** Averaged expression of genes positively contributing to MVS scores on UMAP plot and box plots demonstrating up-regulation of this gene set after vaccination. **c** Average expression of cytotoxicity signature genes on UMAP plot and box plots depicting expression changes of this gene set amongst 11 major immune cell types before and after vaccination.

Supplementary Fig. S4

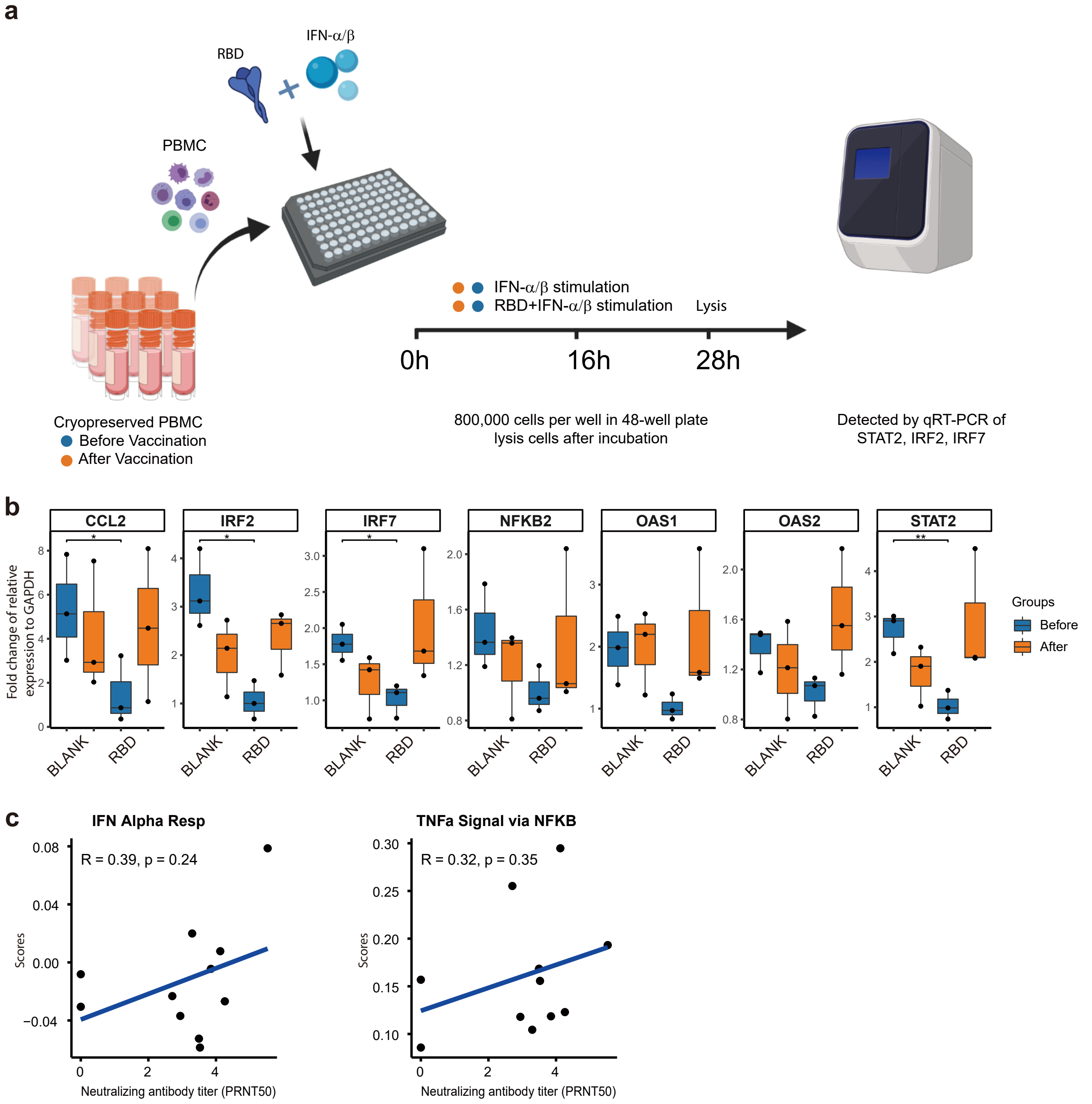

Supplementary Fig. S4 a Schematic overview of experiment. **b** Expression of 7 genes related to type I IFN responses in cultured PBMCs from healthy volunteers before and 28 days after vaccination, with or without RBD pretreatment. (*p<0.05, **p<0.01, n = 3) **c** Pearson's Correlation Coefficient between neutralizing antibody titers and inflammatory responses measured by averaged gene expression of genes associated with TNFα Signaling via NF-κB and with Interferon-α (type I interferon) responses.

Supplementary Fig. S5

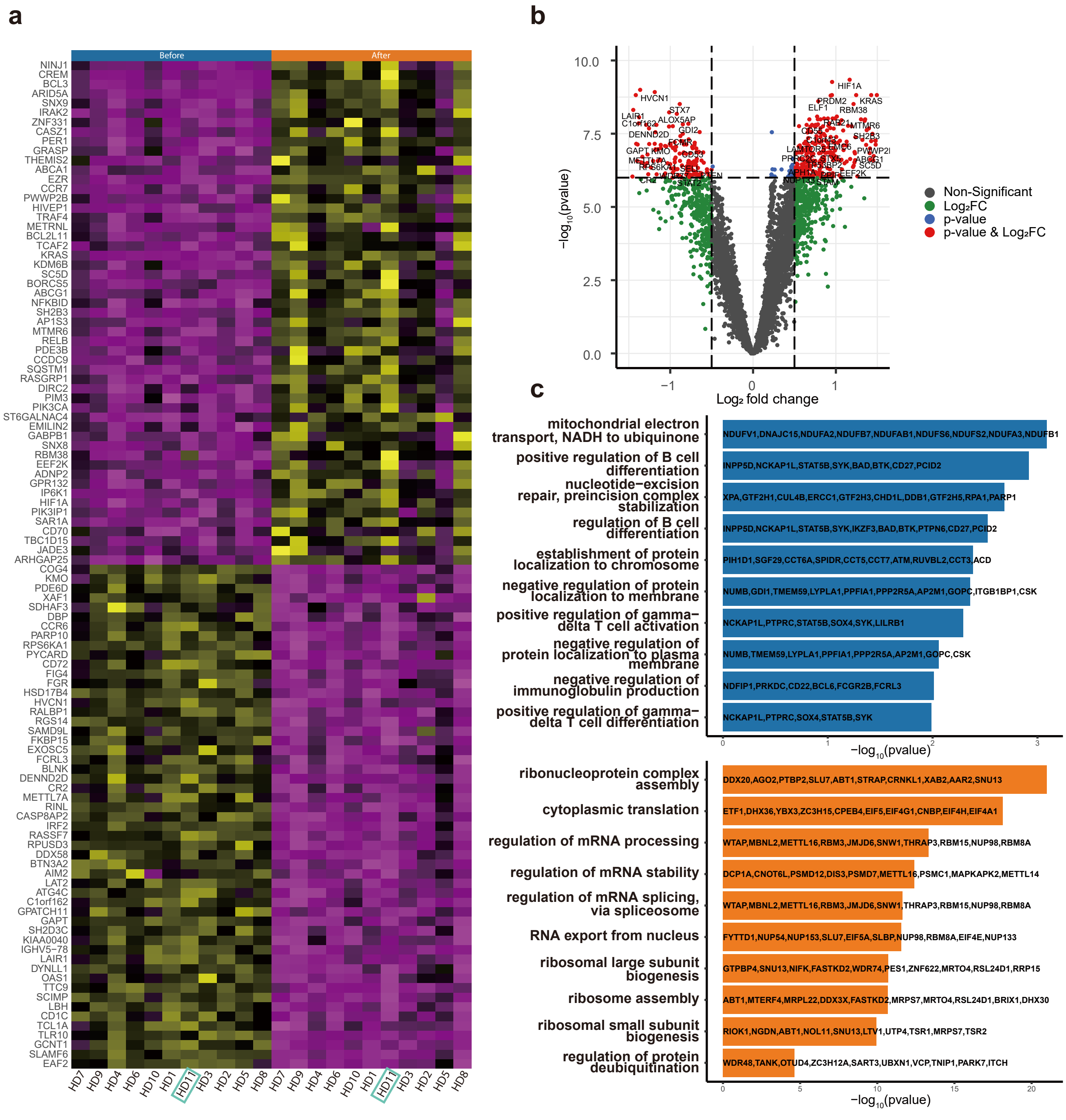

Supplementary Fig. S5 a Differentially expressed genes before and after vaccination in B cells based on scRNA-seq data after pseudo-bulk aggregation. **b** Volcano plot of B-cell specific differentially expressed genes (DEGs) before and after vaccination. **c** Gene Ontology analysis of B-cell specific DEGs.
